# The Diagnosis of Nerve Degeneration

**Published:** 1901-04-20

**Authors:** 


### THE DIAGNOSIS OF NERVE DEGENERATION.

According to a paper by Dr. Mott and Professor Halliburton, recently read before the Royal Society, the marked degeneration of the brain which occurs in general paralysis of the insane is accompanied by the passing of the products of the degeneration into the cerebro-spinal fluid. Nucleo-proteid and choline are the products which can most readily be detected. Choline even can be discovered in the blood, and not only is this the case in general paralysis, but in several other diseases in which degeneration of nervous tissue is a marked feature, such as combined sclerosis, disseminated sclerosis, alcoholic neuritis, and beri-beri. From a series of experiments made on cats, in which the sciatic nerves were divided, it was shown that the presence of these products of degeneration in the blood bore a definite relation to the progress of the degeneration, and it was suggested that the tests by which the presence of these products of nerve degeneration in the blood can be demonstrated might prove of use in the diagnosis of cases in which it was a question whether the symptoms were the outcome of functional or of organic disease.

